# SGLT2 inhibitors for prevention and management of cancer treatment-related cardiovascular toxicity: a review of potential mechanisms and clinical insights

**DOI:** 10.1186/s40959-024-00284-4

**Published:** 2025-02-11

**Authors:** Carl Simela, J Malcolm Walker, Arjun K. Ghosh, Daniel H. Chen

**Affiliations:** 1 University College London Hospital, London, UK; 2 Hatter Cardiovascular Institute, University College London, London, UK; 3 Barts Heart Centre, London, UK; 4 Prince of Wales Hospital, Sydney, NSW Australia; 5 St George Hospital, Sydney NSW, Australia

**Keywords:** SGLT2i, CTR-CVT, Doxorubicin, Trastuzumab, Empagliflozin, Dapagliflozin, Cardiotoxicity

## Abstract

More evidence-based strategies are needed for preventing and managing cancer treatment-related cardiovascular toxicity (CTR-CVT). Owing to the growing body of evidence supporting their cardioprotective role in several cardiac injury scenarios, sodium-glucose cotransporter 2 inhibitors (SGLT2i) may be beneficial for preventing and treating CTR-CVT. In October 2024, a search was conducted of the PubMed database to review full studies investigating the cardioprotective role of SGLT2i against CTR-CVT. We identified 44 full published/pre-print studies and 3 ongoing randomised controlled trial across eight types of cancer treatment (anthracyclines, platinum-containing therapy, immune checkpoint inhibitors, HER2-targeted therapies, kinase inhibitors, androgen deprivation therapies, multiple myeloma therapies and 5-fluorouracil). Most studies used animal models and focussed on primary prevention. 43 of the 44 studies found some cardioprotective effect of SGLT2i against CTR-CVT, which in some cases included preventing ejection fraction decline and aberrations in cardiac electrophysiological parameters. Some studies also observed beneficial effects on mortality. A central triad of anti-inflammatory, anti-oxidative and anti-apoptotic mechanisms likely underlie SGLT2i-mediated cardioprotection against CTR-CVT. Overall, this growing body of research suggests that SGLT2i may be a promising candidate for preventing CTR-CVT either as monotherapy or in combination with other cardioprotective drugs. However, the literature is limited in that no prospective randomised controlled trials investigating SGLT2i for the prevention and management of CTR-CVT exist and most existing human retrospective data is based on diabetic populations. Future work must focus on addressing these limitations of the current literature.

**Supplementary Information:**

The online version contains supplementary material available at 10.1186/s40959-024-00284-4.

## Background

Successful developments in cancer treatment over the recent decades have led to significant improvements in cancer survivorship [1]. However, this success is increasingly tempered by cardiovascular toxicity [2]. Cancer treatment-related cardiovascular toxicity (CTR-CVT) encompasses a broad spectrum of presentations, both acute and chronic, including heart failure with either preserved or reduced ejection fraction (cancer therapy-related cardiac dysfunction or CTRCD), myocarditis, vascular toxicities, acute coronary syndromes, hypertension, cardiac arrhythmias, pericardial disease, and valvular disease. Many mechanisms underlying CTR-CVT have been proposed including oxidative stress and mitochondrial dysfunction [3].

CTR-CVT confers significant mortality and morbidity, whilst also leading to interruptions and/or premature termination of cancer treatment with subsequent negative impacts on cancer outcomes [4, 5]. Currently, there are few pharmacological options for preventing CTR-CVT. Therefore, recent efforts have focused on novel primary and secondary preventative strategies, including exercise [6–8] and pharmacological treatment with Renin Angiotensin Aldosterone System Inhibitors, beta blockers and statins [9–12]; all with varying success.

Sodium-glucose cotransporter 2 inhibitors (SGLT2i) are a class of antidiabetic medication that inhibit the SGLT2 protein in the proximal convoluted tubule resulting in increased glucose excretion [13]. There is minimal SGLT2 protein expression in cardiac tissue. However, a cardioprotective role for SGLT2i has been established in patients with and without diabetes in the setting of reduced, mid-range and preserved ejection fraction [14–17]. This has been attributed to off-target effects whereby SGLT2i interact with other molecular pathways [18]. However, the role of SGLT2i in cardioprotection from CTR-CVT has not yet been widely explored.

We searched the PubMed database to review the current literature published before 8th October 2024 investigating SGLT2 inhibitors for CTR-CVT prevention and treatment. We have considered this in the context of all cancer treatment types with the potential for CTR-CVT documented in the European Society of Cardiology 2022 cardio-oncology guidelines [19]. Pre-print articles and ongoing clinical trials were included. Abstracts and posters were excluded. An additional file summarises the literature search strategy and full list of all cancer treatments included in the literature search [see Additional File 1**]**. Numerical data was extracted from graphs using WebPlotDigitizer software (≈ indicates data that has been extracted with this software) [20].

## Summary of studies

**Fig. 1:**
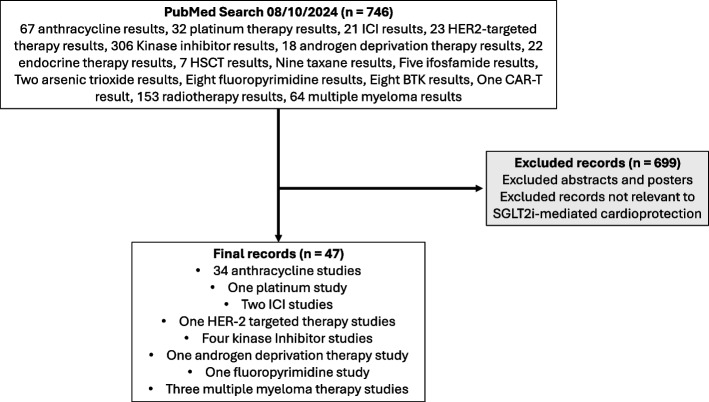
A flowchart illustrating the literature search and literature screening process. No date restriction was used for the literature search. *BTK* = *Bruton Tyrosine Kinase Inhibitor; CAR-T* = *chimeric antigen receptor T-cell; HER-2* = *human epidermal growth factor receptor 2; HSCT* = *haematopoietic stem cell transplantation; ICI* = *immune checkpoint inhibitor*

47 individual records investigating SGLT2i for CTR-CVT were identified (Fig. 1). 34 of the 47 records focused on CTR-CVT due to anthracycline treatment (Tables 3 and 2). Four studies focussed on CTR-CVT due to platinum-containing therapy, HER2-targeted therapy, androgen deprivation therapy, and fluoropyrimidine therapy. Two studies considered immune checkpoint inhibitor therapy. Four studies focussed on CTR-CVT in the context of kinase inhibitor therapy and three studies focussed on CTR-CVT due to multiple myeloma therapy (carfilzomib and cyclophosphamide) (Table 1).

**Table 1:** A summary of 47 individual records (43 published full research articles and three active clinical trials) considering SGLT2 inhibition as cardioprotection and/or treatment against cancer treatment-related cardiovascular toxicity

Anticancer therapy	Studies investigating SGLT2i for primary prevention of CTR-CVT		Studies investigating SGLT2i for treatment of CTR-CVT	
	**Pre-clinical**	**Clinical**	**Pre-Clinical**	**Clinical**
**Anthracyclines**	[24, 30–36, 49–54, 56–62]	[39, 42–46, 104] PROTECT RCT—NCT06341842* EMPACT RCT—NCT05271162* PROTECTAA RCT—NCT06304857*	-	[46–48, 105]
**Platinum-containing therapies**	[67]	-	-	-
**Immune checkpoint inhibitors**	[73]	[74]	-	-
**HER-2 targeted therapy**	[78]	PROTECT RCT—NCT06341842*	-	-
**Kinase inhibitors**	[83–86]	_	-	-
**Androgen deprivation therapies**	-	[95]	-	-
**Endocrine therapies**	-	-	-	-
**Haematopoietic stem cell transplantation**	-	-	-	-
**Taxanes**	-	-	-	-
**Ifosfamide**	-	-	-	-
**Arsenic Trioxide**	-	-	-	-
**Fluoropyrimidines**	[93]	-	-	-
**Chimeric antigen receptor therapy**	-	-	-	-
**Radiotherapy**	-	-	-	-
**Multiple myeloma therapies**	[89–91]	-	-	-

34 anthracycline records, one platinum therapy record, two immune checkpoint inhibitor record, one HER-2 targeted therapy record, four kinase inhibitor records, one androgen deprivation therapy record, one fluoropyridine record, three multiple myeloma therapy record

*RCT* randomised controlled trial

^*^Currently recruiting

*- denotes gaps in the literature*

## SGLT2I for anthracycline-related CTR-CVT

Anthracyclines are highly effective for the treatment of several solid and haematological malignancies. However, adverse cardiovascular effects can occur in approximately 5—10% of anthracycline-treated patients, with the most frequently observed cardiotoxicity being anthracycline-induced cardiomyopathy [21]. This manifests as significant left ventricular ejection fraction (LVEF) decline and overt heart failure (HF), which can happen during treatment or as a late-effect after treatment cessation [22]. Reactive oxygen species and topoisomerase 2β have been implicated as central mediators [23].

21 of the 34 anthracycline-related studies identified were pre-clinical studies using cell or rodent models of CTR-CVT; the remaining 13 studies were in humans – this included three ongoing human randomised controlled trials (Table 2). Doxorubicin was the anthracycline used in all but one of the published studies. The cumulative doxorubicin dose in the rodent studies ranged from 7 mg/kg to 25 mg/kg. All but one of the studies demonstrated a beneficial effect associated with SGLT2 inhibition [24]. This was also the only study that used pirarubicin, which potentially has a different mechanism of myocardial injury compared to doxorubicin-induced cardiotoxicity [25–28].

**Table 2:** Summary of the clinical literature on SGLT2 inhibition as cardioprotection against anthracycline-related cardiovascular toxicity

First Author/Study	Anthracycline Type and Mean Cumulative Dose	Study design	Participants	SGLT2i used	Timing of SGLT2i	Main Effect(s) of SGLT2i
**Gongora et al. 2022 **[44]	Mostly DOX	Retrospective	32 diabetic patients treated with SGLT2i during anthracycline cancer treatment (cases) vs 96 matched controls	EMPA (50%), CANA (34%), DAPA (16%)	Prior to or at point of anthracycline treatment	Reduced overall mortality Reduced heart failure admission, incidence and exacerbation Reduced new cardiomyopathy Reduced clinically significant arrhythmia
**Giangiacomi et al. 2023** [47]	> 250 mg/m^2^ of doxorubicin or equivalent	Case series	7 non-diabetic patients treated with SGLT2i for symptomatic CTRCD	DAPA 10 mg (86%) or EMPA 10 mg (14%) for a median of 24 weeks	After onset of symptomatic CTRCD and at least 6 months after treatment with two other HF drugs	Improved LVEF and improved NYHA Functional Class
**Abdel-Qadir et al. 2023** [43]^**+**^	DOX (77.5%), Epirubicin (14.0%)	Retrospective	99 T2DM patients vs 834 non-SGLT2i controls	DAPA, EMPA, CANA	Prior to or at the point of anthracycline initiation	Reduced HF hospitalisations
**Avula et al. 2024 **[46]^**+**^	DOX (14.4%), Idarubicin (1.6%), Liposomal DOX (1.6%), Daunorubicin (1.6%)	Retrospective	640 T2DM patients on GDMT inc SGLT2i vs 640 T2DM patients on GDMT without SGLT2i	DAPA, EMPA, CANA	-	Reduced mortality, reduced acute HF exacerbations, reduced all-cause mortality, reduced all-cause reduced hospitalisations, reduced emergency-department visits, reduced atrial fibrillation, reduced acute kidney injury, reduced renal replacement therapy
**Hwang et al. 2023** [104]^** +**^	DOX (95%), Epirubicin (1%), DOX + Epirubicin (4%)	Retrospective	780 T2DM patients vs 77,337 non-DM controls and 3,455 T2DM non-SGLT2i controls	-	Prior to or at the point of anthracycline initiation	Reduced composite of heart failure hospitalisation, acute myocardial infarction, ischemic stroke, and death
**Chiang et al. 2023** [45]^**+**^	Anthracycline (8%)	Retrospective	878 SGLT2i-treated T2DM patients vs 878 T2DM non-SGLT2i treated controls	DAPA, EMPA, CANA	After cancer diagnosis	Reduced incident HF hospitalisation, improved survival
**Oda et al. 2024** [48]	DOX 691 mg/m^2^	Case report	69-year-old female	EMPA 10 mg daily	After CTRCD diagnosis	Stabilisation/improvement of LVEF
**Henson et al. 2024** [105]	Anthracyclines	Retrospective	1323 SGLT2i-treated patients with HF + prior AC treatment (61% DM) vs 1323 non-SGLT2i treated patients with HF + prior AC treatment (64% DM)	SGLT2i	-	Reduced all-cause mortality
**Fath et al. 2024** [42]	DOX (86%), (5.9%), Epirubicin (4.4%), Idarubicin (3.8%), Valrubicin (1.4%), Mitoxantrone (1.4%)	Retrospective	706 SGLT2i-treated patients (91% DM) vs 706 non-SGLT2i treated patients	EMPA (57%), CANA (31%), DAPA (26%), ERTU (1.4%)	-	Reduced new-onset HF incidence Reduced HF exacerbations Reduced new-onset arrhythmia
**Daniele et al. 2024** [39]	DOX 317 mg/m^2^	Prospective study	38 SGLT2i-treated breast cancer patients (87% DM) vs 38 non-SGLT2i treated breast cancer patients	EMPA 10 mg daily	Started 7 days before DOX for 6 months	Reduced decline in LVEF over 6 months Reduced GLS impairment

*AC* anthracycline, *CANA* canagliflozin, *CKD* Chronic Kidney Disease, *CTRCD* cancer therapy-related cardiac dysfunction, *DAPA* dapagliflozin, *DM* diabetes mellitus, *DOX* doxorubicin, *EMPA* empagliflozin, *ERTU* Ertugliflozin, *HF* heart failure, *iPSC-CM* induced pluripotent stem cell-derived cardiomyocytes, *LVEF* Left ventricular ejection fraction

– = not specified in original article or not applicable

^+^ other anticancer therapies given

### Cardiac dysfunction

SGLT2i administration before or at the time of anthracycline treatment was associated with an attenuation in anthracycline-induced LVEF decrease in both pre-clinical studies (Table 3) and clinical studies (Table 2). This is in concordance with a recent meta-analysis of 8 rodent studies, which found a statistically significant cardioprotective effect of prophylactic SGLT2 inhibitor use on LVEF (76.9% ± 3.4 vs 61.7% ± 5.8 in controls; *p* < 0.001) in animals receiving anthracycline treatment [29]. Rodent studies also suggest SGLT2 inhibitors attenuate anthracycline-induced impairments in LV strain [30–36]. This is important as LV strain impairments detect early subclinical myocardial injury and have been shown to be a predictor of CTR-CVT [37, 38]. Similarly, in the first prospective human study in this area, Daniele et al. [39] showed a six-month course of empagliflozin started seven days before commencement of high-dose doxorubicin for breast cancer significantly reduced the risk of CTRCD within six months (RR 0.18; 95% CI 0.04 to 0.75). This remained significant even after adjusting for the increased beta-blocker use in the SGLT2i-treated group (adjusted OR of CTCD in control group vs SGLT2i-treated group: 10.29; *p* = 0.0055), thus suggesting a role for SGLT2i for primary prevention against CTRCD. It must be noted however that all patients in this study had either stable heart failure or diabetes mellitus prior to anticancer treatment and had a high or very high baseline risk of CTRCD using the HFA-ICOS score [40]. The ongoing clinical trials (Table 2) will help address whether SGLT2i for primary prevention of CTRCD extends to patients with a lower baseline risk and patients without diabetes.

**Table 3:** Summary of the pre-clinical literature on SGLT2 inhibition as cardioprotection against anthracycline-related cardiovascular toxicity

First Author/Study	Anthracycline Type and Cumulative Dose	Model used	SGLT2i used	Timing of SGLT2i	Main Effect(s) of SGLT2i	Proposed Mechanism(s)
**Sabatino et al. 2020** [35]	DOX 25 mg/kg	C57Bl/6 non-diabetic mice	Oral EMPA 10 mg/kg/day	From the start of DOX treatment	**Attenuated:** Blood pressure drop, LVEF impairment, FS impairment, Longitudinal Strain impairment, Circumferential Strain impairment, diastolic function (E/E’ ratio) impairment	EMPA → reduced ERK activity → ↓ [Na +]_i_ and restored* mitochondrial Ca2 + handling* → reduced cardiac dysfunction
**Oh et al. 2019** [60]	DOX 15 mg/kg	C57Bl/6 non-diabetic mice	EMPA mixed with diet at concentration of 300 mg/kg of diet for 2 weeks	Started 12 h before DOX injection	**Attenuated:** LV mass increase, FS decrease, LVESD increase, myocardial fibrosis increase, cardiac remodelling	SGLT2i → ↑ βOHB → ↓ROS, ↓ apoptotic proteins, ↓ mitochondrial dysfunction → reduced cardiac dysfunction
**Barış et al. 2021** [30]	DOX 18 mg/kg	Non-diabetic Sprague–Dawley rats	Oral EMPA 10 mg/kg every other day	From the start of DOX treatment	**Attenuated:** PR prolongation, QRS amplitude increase, QTc prolongation, LVEDD increase, LVESD increase, LVEF decrease, FS decrease, cardiomyocyte cell size decrease	EMPA → ↓ [Ca^2+^]_i_ → ↓ apoptosis, ↓ myocardial infiltration → reduced cardiac dysfunction*
**Quagliariello et al. 2021** [34]	DOX 15.19 mg/kg	C57Bl/6 non-diabetic mice	Oral EMPA 10 mg/kg/day for 10 days	EMPA started 3 days before DOX	**Attenuated:** FS decrease, LVEF decrease, LS and RS impairments	EMPA → ↓ [Ca^2+^]_i,_ ↓ pro-inflammation, ↓ ROS, ↓ lipid peroxidation, ↓ MyD88 and NLRP3 expression, ↓ ferroptosis and apoptosis, ↓ xanthine oxidase expression → ↓ fibrosis, reduced cardiac dysfunction
**Hu et al. 2023** [52]	DOX 20 mg/kg	C57Bl/6 non-diabetic mice	Oral DAPA 1.5 mg/kg/day for 4 weeks	From the start of DOX treatment	**Attenuated:** LVEF decrease, LVFS decrease, LVIDs decrease, LVIDd decrease	DAPA → ↓ P-p38/P-ERK/TLR4 pathway → ↓ NLRP3 expression → ↓ pro-inflammation → reduced cardiac dysfunction
**Wang et al. 2020 **[36]	DOX 20 mg/kg	Non-diabetic mice	Oral EMPA 0.05 mg/g /day for 5 weeks	Started one week before DOX treatment	**Attenuated:** LVFS decrease, increased cardiac fibrosis, cTnT increase, BNP expression	EMPA → ↑ autophagic flux, → reduced cardiac dysfunction EMPA → ↑ Beclin-1-TLR9-SIRT3 binding → ↑ TLR-9 mitochondrial localisation → ↑ mitochondrial respiration, ↓ DNA damage → reduced cardiac dysfunction
**Chang et al. 2021** [31]	DOX 20 mg/kg	Diabetic STZ Sprague–Dawley rats	Oral DAPA 10 mg/kg/day for 6 weeks (pre-treatment)	DAPA given as a pre-treatment	**Attenuated:** Mortality rate, lower body weight, hyperglycaemia, decreased LVEF, decreased LVFS, increased heart:body weight ratio	DAPA → ↓ ER Stress → ↓ apoptosis, ↑anti-apoptotic protein (Bcl-2) → reduced cardiac dysfunction
**Chang et al. 2022** [32]	DOX 20 mg/kg	Non-diabetic Sprague–Dawley rats	Oral DAPA 10 mg/kg/day for 6 weeks (pre-treatment)	DAPA given as a pre-treatment	**Attenuated:** LVEF decrease, LVFS decrease, increased weight:dry lung weight ratio	DAPA → → ↑anti-apoptotic proteins (Bcl-2 and STAT3) → ↓ apoptosis, ↓ ROS generation → → reduced cardiac dysfunction
**Hsieh et al. 2022 **[33]	DOX 12 mg/kg	Non-diabetic Sprague–Dawley rats	Oral DAPA 0.1 mg/kg/day for 4 weeks	From the start of DOX treatment	**Attenuated:** LVEF decrease, LVFS decrease, cardiac hypertrophy	DAPA → ↑ AKT/PI3K → ↑antioxidants → ↑ ΔΨm, ↓ p-38/NF-κB p65—→ ↓ inflammation, ↓ hypertrophy and ↓ fibrosis → reduced cardiac dysfunction
**Belen et al. 2022** [50]	DOX 15 mg/kg	Non-diabetic albino Sprague–Dawley rats	Oral DAPA 1 mg/kg/day for 15 days	From the start of DOX treatment	**Attenuated:** plasma cTnT increase, plasma pro-BNP increase, fibronectin cardiac expression, plasma TNF-α increase, *cardiomyocyte thickness*	DAPA → ↓ inflammation → ↓ cardiomyocyte fibronectin → reduced cardiac dysfunction
**Shi et al. 2021 **[24]	THP 24 mg/kg	Non-diabetic Sprague–Dawley rats	CANA added to diet at concentration of 60 mg/kg of diet	From the start of THP treatment	No attenuation of THP-induced cardiotoxicity observed	-
**Satyam et al. 2023** [53]	DOX 20 mg/kg	Non-diabetic Wistar rats	Oral DAPA 0.9 mg/kg/day for 8 days	DAPA started before DOX	**Attenuated:** CK-MB increase, increased PR interval, QRS complex amplitude reduction, QTc prolongation	SGLT2i → ↓ oxidative stress, fibrosis, remodelling → reduced cardiac dysfunction*
**Kim et al. 2022 **[49]	DOX 15 mg/kg	Male 8-week-old non-diabetic C57BL/6 J mice	Oral DAPA 1 mg/kg/day daily for 6 weeks	DAPA started one day after DOX	**(Acute) Attenuated:** mortality rate, weight loss, bradycardia** **(Chronic) Attenuated:** LVEF reduction, LV wall thinning, cardiac mass decrease, Stroke volume decrease, LVEDV decrease, LVESV increase**, myocardial atrophy**, myofibrillar thinning and vacuolisation	DAPA + low-dose ARNI → ↑ cardiac fatty acid metabolism, ↑ cardiac GLUT4, ↑ ketogenesis → ↑ β βOHB → ↑ cardiac metabolism → reduced cardiac dysfunction
**Yang et al. 2019** [54]	DOX 7 mg/kg	Non-diabetic adult male Sprague–Dawley rats with CKD	Intraperitoneal EMPA 20 mg/kg/day	EMPA started 24 days after last DOX injection	**Attenuated:** LVESD increase, LVEF decrease, myocardial fibrosis, myocardial gap junction degradation, myocardial apoptosis, DNA damage, reduction in angiogenesis, BNP expression increase, inflammatory marker expression, AT2R expression, oxidative stress	EMPA → ↓ oxidative stress, fibrosis, remodelling, DNA damage → reduced cardiac dysfunction
**Chen 2023** [51]	DOX 20 mg/kg	Non-diabetic adult male Sprague–Dawley rats	Intragastric EMPA 30 mg/kg/day for 4 weeks	From the start of DOX treatment	**Attenuated:** LVESD increase, LVEDD increase, LVEF decrease, myocardial fibre disarrangement, myocardial cell necrosis, serum CK-MB and NT-proBNP increase	EMPA → ↑ antioxidants, ↑ p-AMPK/SIRT-1/PGC-1α levels → reduced myocardial injury → reduced cardiac dysfunction
**Avagimyan et al. 2023** [56]	DOX 15 mg/kg	Inbred male Wistar rats	Oral DAPA 0.9 mg/kg/day for 2 weeks	From the start of DOX treatment	**Attenuated:** Total cholesterol and total triglyceride increase, LDL-cholesterol increase, endothelial dysfunction, cTnI increase, bradycardia, Heart/body weight ratio increase, myocardial inflammation	DAPA → ↑ antioxidants → ↓ vascular inflammation, ↓ endothelial dysfunction, → reduced cardiac damage
**Lin et al. 2024** [62]	DOX 1 μmol/L	Adult male C57BL/6 mouse ventricular myocytes	1 µmol/L EMPA for 30 min	30 min before incubation with DOX	**Attenuated:** Peak shortening decrease, Maximal contraction velocity decrease, Maximal relaxation velocity decrease, Ca^2+^ handling impairments decreased SERCA2a expression, increased NCX1 expression, mitochondrial ROS production	EMPA → ↓ mitochondrial ROS production → ↓ oxidised CaMKII → ↓ RyR2 S2814 phosphorylation → improved calcium handling → reduced cardiac dysfunction
**El-Sawy et al. 2024** [57]	DOX 10 mg/kg	Male mature Swiss albino rats	Oral DAPA 10 mg/kg for 16 days	Pre-treatment for 14 days and two days after DOX	**Attenuated:** Serum CK-MB increase, cardiomyocyte degeneration	DAPA → ↑ HO-1 protein levels, ↓ inflammation and apoptosis ↓ oxidative stress → reduced cardiac damage
**Malik et al. 2024 **[58]	DOX 10 μM	Sprague–Dawley rat cardiomyocytes	EMPA 500 nM for 24 h	Incubated one hour before DOX	**Attenuated:** Apoptosis, oxidative stress, inflammation	EMPA → ↑ STAT3, ↓ ER stress, ↓p-JNK pathway → reduced apoptosis
						EMPA → ↓ TNF- α + IL-10, ↓ ROS → reduced inflammation and oxidative stress
**Quagliariello et al. 2024** [59]	DOX 0.1 – 50 μM DOX 21.7 mg/kg	AC16 Adult Human Cardiomyocytes C57Bl/6 mice	DAPA 10 or 100 nM for 24 h DAPA 10 mg/kg/day for 10 days	Incubated at same time as DOX From start of DOX treatment	**Attenuated:** LVEF decline, FS decline, radial and longitudinal strain impairment	DAPA → ↑ ATP, ↓ mitochondrial dysfunction, ↓ lipid peroxidation, ↓ inflammation, ↓ [Ca^2+^]_i_, ↓ ferroptosis → reduced cardiac dysfunction
**Chang et al. 2024** [61]	DOX 5 μM DOX 20 mg/kg	Human iPSC-CMs C57Bl/6 mice	EMPA 0 – 10 µM Oral EMPA 10 mg/kg/day	24 h pretreatment before DOX incubation From 3 days before DOX treatment	**Attenuated:** LVEF decrease, LVFS decrease	EMPA → ↓ p-JNK, ↓ ROS → ↓ apoptosis → reduced cardiac dysfunction

*ΔΨm* mitochondrial membrane potential*, AC* anthracycline*, ARNI* sacubitril/valsartan, *AT2R* angiotensin II receptor, *[Ca*^*2*+^*]*_*i*_ intracellular calcium concentration, *CANA* canagliflozin, *CKD* Chronic Kidney Disease, *CK-MB* creatine kinase myocardial band, *CTRCD* cancer therapy-related cardiac dysfunction, *DAPA* dapagliflozin, *DM* diabetes mellitus, *DOX* doxorubicin, *EMPA* empagliflozin, *ERTU* Ertugliflozin, *iPSC-CM* induced pluripotent stem cell-derived cardiomyocytes, *LVEF* Left ventricular ejection fraction, *LVFS* Left Ventricular Fractional Shortening, *THP* pirarubicin

^*^not tested experimentally. Italics = non statistically significant changes

– not specified in original article or not applicable

^**^ effect only observed with DAPA + low-dose ARNI together, but not DAPA alone

^+^ other anticancer therapies given

[]_i_ denotes intracellular concentration

### Cardiac morbidity and mortality

Both the pre-clinical and clinical literature suggest a potential beneficial effect of SGLT2i on cardiac morbidity and mortality in the setting of anthracycline treatment (Tables 3 and 2). A recent meta-analysis of four observational clinical studies supported this by showing SGLT2i reduced heart failure hospitalisations (RR: 0.44; 95% CI 0.28 to 0.71; *p* < 0.001), all-cause mortality (RR: 0.35; 95% CI 0.30 to 0.40; *p* < 0.001), as well as clinically significant arrhythmias (RR: 0.40; 95% CI 0.22 to 0.70; *p* = 0.001) in T2DM patients receiving cancer treatments, including anthracyclines and platinum-based chemotherapy [41]. More recently, Fath et al. [42] retrospectively showed that SGLT2i was associated with a reduced incidence of new-onset heart failure and arrhythmias in patients receiving anthracyclines. However, there was no significant benefit on hospitalisation or mortality rates at two-year follow-up. In a similar retrospective clinical study, Abdel-Qadir et al. [43] also found no significant effect on mortality rates, but did find SGLT2i reduced heart failure hospitalisations. A recent small prospective clinical study failed to show a beneficial effect on mortality or heart failure hospitalisations with SGLT2i use [39]. These conflicting data may be due to small sample sizes as well as heterogenous SGLT2i timing between individual studies as some retrospective studies [43, 44] only included patients who started SGLT2i treatment prior to cancer treatment, whilst others [42, 45, 46] did not specify this as an inclusion criterion.

Recent clinical case reports also show a beneficial effect of SGLT2i on LVEF and New York Heart Association Functional Class in patients with established anthracycline-induced heart failure [47, 48]. Avula et al. [46] also showed that guideline-directed medical therapy (GDMT) including SGLT2i resulted in reduced mortality and heart failure exacerbations compared to GDMT without SGLT2i in T2DM patients with CTRCD.

Collectively, this suggests SGLT2i may be valuable for primary and secondary prevention against CTR-CVT, particularly in combination with other cardioprotective medications.

### SGLT2i in combination with other cardioprotective strategies

The potential for additive cardioprotective benefits from combination therapy was explored by Kim et al. [49], who demonstrated that mice treated with low-dose sacubitril/valsartan in combination with dapagliflozin resulted in no significant change to LVEF following exposure to doxorubicin (74.5 ± 1.6% at baseline vs. 73.8 ± 1.8% post doxorubicin; *p* > 0.05) at nine-week follow up. On the contrary, whilst statistical comparisons were not reported, mice treated with either dapagliflozin or sacubitril/valsartan alone appeared to have only had partial protection against LVEF decline. This suggests that combination neurohormonal therapy confers additional cardioprotection above that conferred by SGLT2i monotherapy.

### Mechanisms of cardioprotection

#### Anti-inflammatory mechanisms

Thirteen studies suggested an anti-inflammatory mechanism in SGLT2i-mediated cardioprotection against anthracycline-induced cardiotoxicity (AIC) [30, 33, 34, 50–59]. This is partially mediated through signalling pathways involving PI3K, AKT, Nrf2, p38 and NF-κB as well as several inflammatory cytokines (Table 3). SGLT2i also attenuates doxorubicin-induced cardiomyopathy and the associated pro-inflammatory cytokine storm by downregulating the NLRP3 inflammasome [34, 52, 59]. This is likely driven by downregulation of doxorubicin-induced P-p38, P-ERK, and TLR4 expression.

#### Anti-apoptotic mechanisms

Nine studies [30–32, 34, 54, 57, 58, 60, 61] demonstrated anti-apoptotic activity as a mechanism for SGLT2i-mediated cardioprotection against AIC. This is largely conferred by downregulating pro-apoptotic proteins (such as cleaved caspase 3 and C/EBP Homologous Protein) and upregulating anti-apoptotic proteins (such as Bcl-2), which are upregulated and downregulated respectively by anthracycline treatment (Table 3). Increases in beta-hydroxybutyrate (BOHB) concentration [60], reduced endoplasmic reticulum stress [31, 58], increased *STAT3* expression [32, 58], reduced ferroptosis [34, 59] and reduced *JNK* activity [58, 61] likely also play a role in SGLT2i-mediated apoptosis reduction.

#### Anti-oxidative mechanisms

Several studies showed SGLT2i reduced anthracycline-induced oxidative stress [31–34, 51, 53, 54, 56–58, 60–62], cardiac energy metabolism impairments and mitochondrial function impairments [30, 33, 35, 36, 49, 51, 54, 59, 60]. SGLT2i treatment reduced oxidative stress via increased levels of antioxidants and reduced reactive oxygen species (ROS) production. The PI3K/AKT/Nrf2 and beta-hydroxybutyrate (BOHB) pathways are likely integral to this SGLT2i-mediated antioxidative activity in AIC (Table 3).

However, not all studies support anti-oxidative action as a mechanism for cardioprotection. Barış et al. [30] did not find a difference in oxidative stress levels between any of the study groups. Instead, they propose restored intracellular calcium homeostasis and restored mitochondrial biogenesis are the main mechanisms underlying SGLT2i-mediated cardioprotection in this context.

#### Restoring intracellular ion homeostasis

Studies show that rodent and human cardiomyocytes co-incubated with empagliflozin and doxorubicin have a significantly attenuated elevation in intracellular calcium concentration compared to cardiomyocytes incubated with doxorubicin alone [34, 59, 63]. Lin and colleagues also showed that empagliflozin attenuated doxorubicin-induced reductions in sarcoplasmic reticulum calcium. They showed this may be mediated by SGLT2i attenuating several doxorubicin-induced cellular changes, including decreased SERCA2a expression, increased NCX1 expression and increased oxidation of Ca^2+^/Calmodulin Dependent Protein Kinase II [62] (Table 3).

## SGLT2I for platinum-containing therapy-related CTR-CVT

Platinum-containing chemotherapy can be associated with cardiovascular toxicity including arterial and venous thrombosis, and less commonly, vasospasm [64, 65]. Several mechanisms have been proposed, including oxidative stress and lipid peroxidation [66].

Canagliflozin treatment in rats attenuated several molecular and histopathological features of platinum-containing chemotherapy cardiotoxicity, including cardiac inflammation and apoptosis [67]. This translated to a reduced CK-MB level in canagliflozin and cisplatin treated rats compared to rats treated with cisplatin alone (≈ 25 U/L vs 15 U/L; *p* < 0.05). They propose a triad of anti-inflammatory, antioxidant and anti-apoptotic effects underlie this [67]. However, the CK-MB level in the canagliflozin and cisplatin treated rats was still significantly higher than the control rats (≈ 10 U/L; *p* < 0.05). Additionally, this study did not perform mortality analyses or quantify heart function.

There are currently no human studies investigating platinum-containing chemotherapy, but the study described suggests this may be a useful avenue to explore, possibly to be used in combination with antioxidants [68]. Such a strategy may be particularly useful when started before chemotherapy or in the early phases of treatment as suggested by Cameron et al. [65], who demonstrated endothelial dysfunction in the first 24 h of treatment in patients receiving cisplatin-containing chemotherapy.

## SGLT2I for immune checkpoint inhibitor-related CTR-CVT

Immune-checkpoint inhibitors (ICI), such as ipilimumab and pembrolizumab, prevent tumour-cell mediated downregulation of the T-cell response against neoplastic cells [69]. However, this subsequent immune activation can be associated with off-target effects on several organ systems, including the cardiovascular system [70]. ICI cardiotoxicity can have a broad range of clinical manifestations; whilst arrhythmias are the most common, myocarditis is the most feared cardiac immune-related complication due to its high mortality in up to 50% of patients [71]. Pembrolizumab and ipilimumab confer the highest risks [72].

Quagliariello et al. [73] showed that under high glucose conditions, to model hyperglycaemia in cancer patients, empagliflozin attenuated several deleterious cellular function parameters induced by ipilimumab treatment in AC16 human cardiomyocytes, including reduced cell viability, increased oxidative stress and increased inflammation. This effect was mediated via reduced expression of the NLRP3 inflammasome, which is upregulated in high glucose conditions and has been previously implicated in SGLT2i-mediated cardioprotection against AIC.

In the first retrospective clinical study in this area, Perelman et al. [74] showed that SGLT2i treatment prior to ICI treatment reduced risk of all-cause mortality in cancer patients with diabetes (HR 0.30; 95% CI 0.104 to 0.865; *p* = 0.002). Despite this, there was no effect on any cardiovascular outcome, including MACE, HF admissions and HF exacerbations at a 28-month median follow-up. This may reflect insufficient follow-up duration, small sample sizes or a non-cardiovascular mechanism of reduced all-cause mortality in this SGLT2i-treated cohort, such as improved cancer outcomes.

From both these studies it is unclear whether any beneficial cardioprotective effect would be replicated in normoglycemic conditions, and thus have relevance to non-diabetic patients. Additional studies, both pre-clinical and clinical, are needed to establish this.

## SGLT2I for HER2 therapy-related CTR-CVT

HER2-targeted therapies target the Human epidermal growth factor receptor-2 (HER2), which is expressed in the cardiomyocyte membrane and is needed for normal cardiac function [75]. Therefore, inhibition of HER2 signalling with HER2-targeted therapies can be associated with cardiotoxicity, which typically manifests as left ventricular systolic dysfunction [76]. Unlike anthracycline-induced CTRCD, cardiac dysfunction caused by HER2-targeted therapy is independent of cumulative dose and is a reversible phenomenon in the majority of cases [77].

Only one study investigated the cardioprotective effect of SGLT2i against cardiotoxicity induced by HER2-targeted therapy [78]. This showed that mice treated with empagliflozin and trastuzumab had better systolic function (Fractional Shortening ≈ 33% vs 44%; *p* < 0.05, EF ≈ 70% vs 85%; *p* < 0.05), decreased troponin (≈ 4.0 ng/ml vs 2.5 ng/ml; *p* < 0.05), decreased oxidative stress and decreased mitochondrial damage compared to mice treated with trastuzumab alone. Empagliflozin also protected against trastuzumab-induced lusitropy perturbations by restoring intracellular calcium handling. These trastuzumab-induced deteriorations in cardiac function were nearly completely prevented with empagliflozin treatment.

In addition to restoring calcium homeostasis, Min and colleagues proposed three main mechanisms of empagliflozin’s cardioprotective effect: anti-apoptotic processess, anti-oxidative processes and anti-ferroptosis [78]. This is in agreement with a growing body of research supporting the use of antioxidant therapy to prevent HER2-targeted therapy induced cardiotoxicity [79].

## SGLT2I for kinase inhibitor-related CTR-CVT

Kinase inhibitors are a broad group of molecules used for treating solid and haematological malignancies, including metastatic breast cancer and mantle cell lymphoma [80]. Kinase inhibitors are associated with several cardiovascular toxicities, including atrial arrhythmia and heart failure [81]. Off-target inhibition of cardiac kinases causing mitochondrial dysfunction and apoptosis likely underlies this [82].

We identified four pre-clinical studies investigating the efficacy of SGLT2i to prevent cardiovascular toxicity induced by kinase inhibitors [83–86]. Cardioprotective effects of SGLT2i on a cellular level included reduced sorafenib-induced apoptosis and an attenuation of ponatinib-induced reductions in human aortic endothelial cell viability [83, 86]. This is important as endothelial dysfunction is a precursor to several pathophysiological cardiovascular processes and toxicities [87, 88]. Empagliflozin treatment also attenuated several ponatinib and sunitinib-induced deteriorations in echocardiographic parameters in mice, including LVEF decline [84, 85]. Ren et al. [84] also showed improved blood pressure and coronary flow reserve in sunitinib-treated animals treated with SGLT2i. Whilst most sunitinib-induced cardiac impairments were completely prevented when empagliflozin was co-administered with the sunitinib, LVEF was still significantly lower than untreated controls (76.18% vs 84.92%; *p* < 0.05), thus showing empagliflozin conferred only partial protection. Cell death, cell viability and apoptotic cells showed a similar phenomenon. It is unclear whether combined treatment with other cardioprotective drugs, an increased SGLT2i dose or a SGLT2i pre-treatment phase before sunitinib administration, would completely prevent these impairments.

Empagliflozin also increased autophagic flux, which had been reduced by ponatinib [83, 85]. Ren et al. [84] had similar findings in the context of sunitinib-induced cardiotoxicity. Reduced inflammation, reduced ferroptosis and reduced DNA damage were also implicated in SGLT2i-mediated reduction of sorafenib-induced cardiotoxicity [86].

## SGLT2I for CTR-CVT caused by other anticancer therapies

Three studies were identified investigating SGLT2i for CTR-CVT caused by the multiple myeloma therapies carfilzomib [89, 90] and cyclophosphamide [91]. Using an in-vitro model, Dabour et al. [89] showed that an AMPK-dependent pathway likely underlies canagliflozin-mediated reductions in carfilzomib-induced endothelial cell apoptosis. George et al. [90] showed empagliflozin reduced cardiomyocyte histological changes by reducing endoplasmic reticulum stress, inflammation, oxidative stress and apoptosis caused by carfilzomib. Neither study investigated impacts on cardiac function or mortality.

Mahmoud Refaie et al. [91] found several amelioratory effects of dapagliflozin on cyclophosphamide-induced cardiotoxicity, including reduced blood pressure and reduced myocardial oedema. On a cellular level, dapagliflozin prevented cyclophosphamide-induced reductions in total antioxidant capacity.

5-FU is a fluoropyrimidine used for solid tumours that is classically associated with up to 9% risk of myocardial ischaemia secondary to coronary vasospasm caused by endothelial and smooth muscle dysfunction [92]. Refaie et al. [93] showed that oral empagliflozin mitigated hypertension, troponin I elevation and heart weight increase in male Wistar rats following one toxic single intraperitoneal dose of 5-FU. Similarly to other findings discussed herein, anti-apoptotic, anti-inflammatory and anti-oxidative mechanisms were implicated. There are currently no other studies investigating SGLT2i for preventing 5-FU cardiotoxicity.

Whilst androgen deprivation therapy is an important aspect of contemporary prostate cancer treatment, it is associated with increased cardiovascular risk resulting from the loss of cardioprotection conferred by physiological levels of circulating male sex hormone [94]. Tang et al. [95] found SGLT2i treatment within the 12 months preceding gonadotropin-releasing hormone (GnRH) agonist commencement was associated with reduced MACE (HR 0.55; 95% CI: 0.35 to 0.87), reduced heart failure (HR 0.57; 95% CI: 0.34 to 0.94), reduced myocardial infarction (HR 0.23; 95% CI 0.08 to 0.70) and reduced all-cause mortality (HR 0.34; 95% CI 0.19 to 0.61) over two years in patients with diabetes. Interestingly, there was no protective effect on atrial fibrillation (HR 0.96; 95% CI 0.38 to 2.42), which is in concordance with recent a meta-analysis [96] and may represent the older age of patients in this study who are more likely to have extensive atrial remodelling that is less amenable to rhythm control therapy. Nonetheless, Tang and colleagues’ other beneficial findings are promising and should be interrogated further with additional clinical studies.

## Additional considerations

### Safety

SGLT2 inhibitors are likely safe and tolerable as none of the human cohort studies reviewed herein reported a statistically significant increased risk of important adverse effects, such as ketoacidosis or urinary infections. Pre-clinical studies also suggest SGLT2i does not attenuate the anticancer effects of anthracyclines [97], platinum-based therapies [98], ICIs [73], endocrine therapies [99], taxane therapies [100] and multiple myeloma therapies [90]. There is also growing clinical evidence suggesting SGLT2i have an independent anticancer effect that has a synergistic role with established cancer treatments and may improve cancer-specific survival [101, 102]. However, further prospective trials are needed to confirm both these points.

### Literature limitations

The literature is heterogenous in design, which makes it difficult to draw conclusions applicable to clinical practice. Additionally, few pre-clinical studies made a comparison between post-SGLT2i treatment parameters and baseline parameters, thus making it difficult to confirm complete normalisation of cardiac function and outcomes with SGLT2i treatment. Furthermore, most of the literature focusses on animal models that use different surrogate endpoints, so it is unclear if this translates into clinically relevant treatment benefits for cancer patients. The relative paucity of negative studies may also reflect a publication bias.

The clinical studies described above must be interpreted cautiously, particularly as T2DM patients represented a large proportion of the SGLT2i-treated cohorts (Table 2). Out of the human studies reviewed herein, only the two anthracycline case studies exclusively considered SGLT2i in individuals without T2DM [47, 48]. It is therefore unclear whether SGLT2i-bsaed CTR-CVT primary and secondary prevention will also be beneficial in non-diabetic individuals, particularly as cardiomyocyte *SGLT2* expression increases in T2DM [103]. It is also difficult to exclude the antidiabetic mechanism of SGLT2i as the primary driver behind positive clinical findings in T2DM patients.

## Conclusion

**Fig. 2:**
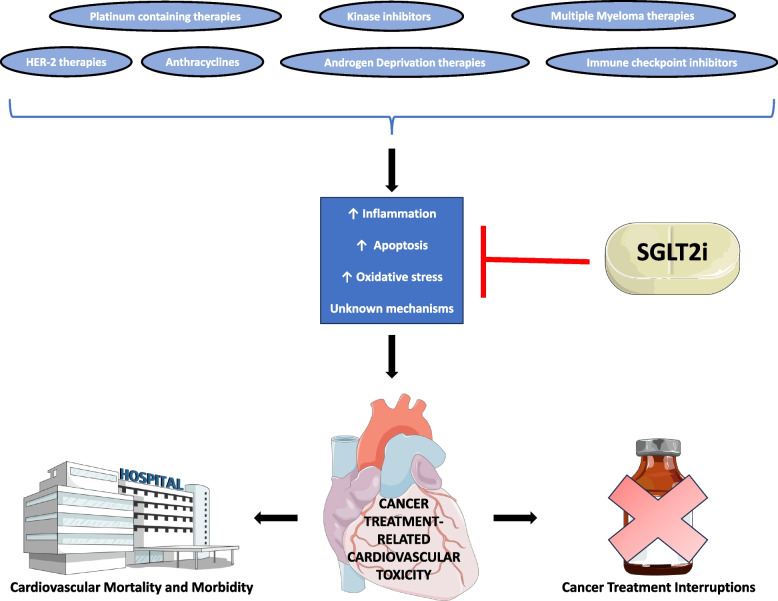
An illustrative summary of the literature. SGLT2i mitigate the apoptotic, oxidative and inflammatory pathways underlying cancer treatment-related cardiovascular toxicity. This should reduce cancer treatment interruptions and cardiovascular mortality/morbidity. *SGLT2i* = *sodium-glucose cotransporter 2 inhibitor*

SGLT2i may be a viable primary and secondary prevention strategy for CTR-CVT caused by several anticancer therapies, particularly anthracyclines (Fig. 2). However, much of the current evidence base is derived from retrospective studies in T2DM populations, thus raising the question of whether cardioprotection extends to patients without diabetes. Additionally, no studies have been published for several cancer therapies, including Bruton Tyrosine Kinase inhibitors and CAR-T therapy [see Additional File 1 for full literature search]. Given that a triad of anti-inflammatory, antioxidative and anti-apoptotic mechanisms may be the unifying mechanisms underlying SGLT2i-mediated cardioprotection, SGLT2i may be beneficial for preventing toxicities induced by additional cancer therapies, particularly those involving inflammation, such as CAR-T related inflammatory responses. Several potential drugs may be useful in combination with SGLT2i for preventing CTR-CVT. This might include well-established cardioprotective therapy, such as RAAS inhibitors, to novel compounds, such as SIRT3 activators. Hypothesis-generating pre-clinical studies as well as prospective clinical trials are needed to establish these points further.

## Supplementary Information

Additional file 1. Complete literature search strategy with search teams used and number of records identified.

## Data Availability

No datasets were generated or analysed during the current study.

## Abbreviations

(P)-ERK: (Phospho) - Extracellular signal-regulated kinases
AIC: anthracycline induced cardiotoxicity
AMPK: AMP - activated protein kinase
ATP: adenosine triphosphate
BOHB: beta-hydroxybutyrate
C/EBP: CCAAT/enhancer binding proteins
CANA: canagliflozin
CTRCD: cancer therapy-related cardiac dysfunction
CTR-CVT: cancer treatment-related cardiovascular toxicity
DAPA: dapagliflozin
DOX: doxorubicin
EMPA: empagliflozin
FS: fractional shortening
HER-2: human epidermal growth factor receptor 2
HO-1: Heme -oxygenase 1
ICI: immune checkpoint inhibitor
LVEDV: left ventricular end diastolic volume
LVEF: Left Ventricular Ejection Fraction
LVESD: left ventricular end systolic diameter
MAC(C)E: Major adverse cardiac (and cerebrovascular) event
mTORC1: mammalian target of rapamycin complex 1
NLRP3: NOD-, LRR- and pyrin domain-containing protein 3
NYHA: New York Heart Association
ROS: reactive oxygen species
SGLT2i: sodium-glucose cotransporter 2 inhibitor
SIRT3: Sirtuin 3
T2DM: Type 2 Diabetes Mellitus
TLR4: Toll-like receptor 4
