# Supplementary material for: SGLT2 inhibitors for prevention and management of cancer treatment-related cardiovascular toxicity: a review of potential mechanisms and clinical insights
Source: Cardiooncology. 2025 Feb 11;11:15. doi: 10.1186/s40959-024-00284-4 (PMC11818010; doi:10.1186/s40959-024-00284-4)
Supplement: Supplementary file 1 — Additional file 1. Complete literature search strategy with search teams used and number of records identified. [file 40959_2024_284_MOESM1_ESM.pdf]

Additional File 1

PubMed Search Strategy

SGLT2 inhibitors in anthracycline induced cardiotoxicity

((Anthracyclines) OR (anthracycline) OR (daunorubicin) or (doxorubicin) OR (idarubicin) OR (mitoxantrone) OR (epirubicin) OR (valrubicin)) AND ((empagliflozin) OR (dapagliflozin) OR (SGLT2i) OR (Sodium-Glucose) OR (canagliflozin) OR (Ipragliflozin) OR (Sodium-Glucose Cotransporter) OR (Luseogliflozin) OR (SGLT2) OR (ertugliflozin) OR (tofogliflozin) OR (remogliflozin) OR (sodium glucose cotransporter 2)) – 08/10/2024 – 67 results, 34 relevant (21 pre-clinical published studies, ten published clinical studies and three ongoing randomised controlled trials)

SGLT2 inhibitors in platinum-containing therapy-induced cardiotoxicity

((Platinum) OR (Platinum chemotherapy) OR (Platinum-containing therapy) OR (Cisplatin) OR (carboplatin) OR (Oxaliplatin)) AND ((empagliflozin) OR (dapagliflozin) OR (SGLT2i) OR (Sodium-Glucose) OR (canagliflozin) OR (Ipragliflozin) OR (Sodium-Glucose Cotransporter) OR (Luseogliflozin) OR (SGLT2) OR (ertugliflozin) OR (tofogliflozin) OR (remogliflozin) OR (sodium glucose cotransporter 2)) – 08/10/2024, 32 results, one relevant

SGLT2 inhibitors in immune checkpoint inhibitor-induced cardiotoxicity

((immune checkpoint) OR (ipilimumab) OR (tremelimumab) OR (nivolumab) OR (cemiplimab) OR (pembrolizumab) OR (atezolizumab) OR (avelumab) OR (durvalumab)) AND ((empagliflozin) OR (dapagliflozin) OR (SGLT2i) OR (Sodium-Glucose) OR (canagliflozin) OR (Ipragliflozin) OR (Sodium-Glucose Cotransporter) OR (Luseogliflozin) OR (SGLT2) OR (ertugliflozin) OR (tofogliflozin) OR (remogliflozin) OR (sodium glucose cotransporter 2)) – 08/10/2024, 21 results, two relevant

SGLT2 inhibitors in HER2 targeted therapy-induced cardiotoxicity

((HER-2) OR (HER2) OR (Human epidermal receptor 2) OR (Herceptin) OR (Trastuzumab) OR (pertuzumab) OR (trastuzumab emtansine) OR (neratinib) OR (HER-2 targeted) OR (erbB2)) AND ((empagliflozin) OR (dapagliflozin) OR (SGLT2i) OR (Sodium-Glucose) OR (canagliflozin) OR (Ipragliflozin) OR (Sodium-Glucose Cotransporter) OR (Luseogliflozin) OR (SGLT2) OR (ertugliflozin) OR (tofogliflozin) OR (remogliflozin) OR (sodium glucose cotransporter 2)) – 08/10/2024, 23 results, one relevant

SGLT2 inhibitors in kinase inhibitor-induced cardiotoxicity

((dasatinib) OR (nilotinib) OR (ponatinib) OR (sunitinib) OR (multi targeted kinase) OR (bosutinib) OR (TKI) OR (tyrosine kinase) OR (tyrosine kinase inhibitor) OR (alpelisib) OR (PI3K) OR (Phosphoinositide 3-kinase inhibitor) OR (idelalisib) OR (umbralisib) OR (duvelisib)) AND ((empagliflozin) OR (dapagliflozin) OR (SGLT2i) OR (Sodium-Glucose) OR (canagliflozin) OR (Ipragliflozin) OR (Sodium-Glucose Cotransporter) OR (Luseogliflozin) OR (SGLT2) OR (ertugliflozin) OR (tofogliflozin) OR (remogliflozin) OR (sodium glucose cotransporter 2)) – 08/10/2024, 182 results, three relevant

SGLT2 inhibitors in androgen deprivation therapy-induced cardiotoxicity

((GnRH agonist) OR (goserelin) OR (histrelin) OR (leuprorelin) OR (triptorelin) OR (GnRH antagonist) OR (Degarelix) OR (Relugolix) OR (antiandrogen) OR (Bicalutamide) OR (Flutamide) OR (Nilutamide) OR (androgen deprivation therapy) OR (Apalutamide) OR (Darolutamide) OR (Enzalutamide) OR (androgen inhibitor) OR (Abiraterone)) AND ((empagliflozin) OR (dapagliflozin) OR (SGLT2i) OR (Sodium-Glucose) OR (canagliflozin) OR (Ipragliflozin) OR (Sodium-Glucose Cotransporter) OR (Luseogliflozin) OR (SGLT2) OR (ertugliflozin) OR (tofogliflozin) OR (remogliflozin) OR (sodium glucose cotransporter 2)) – 08/10/2024, 18 results, one relevant

SGLT2 inhibitors in endocrine therapy-induced cardiotoxicity

((Selective oestrogen receptor modulator) OR (SERM) OR (selective estrogen receptor modulator) OR (tamoxifen) OR (toremifene) OR (Aromatase inhibitor) OR (Letrozole) OR (Anastrozole) OR (Exemestane)) AND ((empagliflozin) OR (dapagliflozin) OR (SGLT2i) OR (Sodium-Glucose) OR (canagliflozin) OR (Ipragliflozin) OR (Sodium-Glucose Cotransporter) OR (Luseogliflozin) OR (SGLT2) OR (ertugliflozin) OR (tofogliflozin) OR (remogliflozin) OR (sodium glucose cotransporter 2)) – 08/10/2024, 22 results, zero relevant

SGLT2 inhibitors in Cyclin-dependent kinase 4/6 inhibitor-induced cardiotoxicity

((CDK4/6 inhibitor) OR (Palbociclib) OR (Ribociclib) OR (abemaciclib)) AND ((empagliflozin) OR (dapagliflozin) OR (SGLT2i) OR (Sodium-Glucose) OR (canagliflozin) OR (Ipragliflozin) OR (Sodium-Glucose Cotransporter) OR (Luseogliflozin) OR (SGLT2) OR (ertugliflozin) OR (tofogliflozin) OR (remogliflozin) OR (sodium glucose cotransporter 2)) – 08/10/2024, one result, zero relevant

SGLT2 inhibitors in anaplastic lymphoma kinase inhibitor-induced cardiotoxicity

((Anaplastic lymphoma kinase inhibitor) OR (ALK inhibitor) OR (crizotinib) OR (brigatinib) OR (lorlatinib) OR (alectinib) OR (ceritinib)) AND ((empagliflozin) OR (dapagliflozin) OR (SGLT2i) OR (Sodium-Glucose) OR (canagliflozin) OR (Ipragliflozin) OR (Sodium-Glucose Cotransporter) OR (Luseogliflozin) OR (SGLT2) OR (ertugliflozin) OR (tofogliflozin) OR (remogliflozin) OR (sodium glucose cotransporter 2)) – 08/10/2024, one result, zero relevant

SGLT2 inhibitors in epidermal growth factor receptor inhibitor-related cardiotoxicity

((Epidermal growth factor receptor inhibitor) OR (Osimertinib)) AND ((empagliflozin) OR (dapagliflozin) OR (SGLT2i) OR (Sodium-Glucose) OR (canagliflozin) OR (Ipragliflozin) OR (Sodium-Glucose Cotransporter) OR (Luseogliflozin) OR (SGLT2) OR (ertugliflozin) OR (tofogliflozin) OR (remogliflozin) OR (sodium glucose cotransporter 2)) – 08/10/2024, 27 results, zero relevant

SGLT2 inhibitors in haematopoietic stem cell transplantation-related cardiotoxicity

((haematopoietic stem cell transplantation) OR (HSCT) OR (autologous) OR (asct)) AND ((empagliflozin) OR (dapagliflozin) OR (SGLT2i) OR (Sodium-Glucose) OR (canagliflozin) OR (Ipragliflozin) OR (Sodium-Glucose Cotransporter) OR (Luseogliflozin) OR (SGLT2) OR (ertugliflozin) OR (tofogliflozin) OR (remogliflozin) OR (sodium glucose cotransporter 2)) – 08/10/2024, seven results, zero relevant

SGLT2 inhibitors in taxane-related cardiotoxicity

((taxane) OR (paclitaxel) OR (docetaxel)) AND ((empagliflozin) OR (dapagliflozin) OR (SGLT2i) OR (Sodium-Glucose) OR (canagliflozin) OR (Ipragliflozin) OR (Sodium-Glucose Cotransporter) OR (Luseogliflozin) OR (SGLT2) OR (ertugliflozin) OR (tofogliflozin) OR (remogliflozin) OR (sodium glucose cotransporter 2)) – 08/10/2024, nine results, zero relevant

SGLT2 inhibitors in ifosfamide-related cardiotoxicity

((ifosfamide)) AND ((empagliflozin) OR (dapagliflozin) OR (SGLT2i) OR (Sodium-Glucose) OR (canagliflozin) OR (Ipragliflozin) OR (Sodium-Glucose Cotransporter) OR (Luseogliflozin) OR (SGLT2) OR (ertugliflozin) OR (tofogliflozin) OR (remogliflozin) OR (sodium glucose cotransporter 2)) – 08/10/2024, five results, zero relevant

SGLT2 inhibitors in arsenic trioxide-related cardiotoxicity

((Arsenic trioxide)) AND ((empagliflozin) OR (dapagliflozin) OR (SGLT2i) OR (Sodium-Glucose) OR (canagliflozin) OR (Ipragliflozin) OR (Sodium-Glucose Cotransporter) OR (Luseogliflozin) OR (SGLT2) OR (ertugliflozin) OR (tofogliflozin) OR (remogliflozin) OR (sodium glucose cotransporter 2)) – 08/10/2024, two results, zero relevant

SGLT2 inhibitors in FMS-like tyrosine kinase 3 inhibitor-related cardiotoxicity

((FMS-like tyrosine kinase 3 inhibitor) OR (FLT3) OR (midostaurin) OR (gilteritinib))AND ((empagliflozin) OR (dapagliflozin) OR (SGLT2i) OR (Sodium-Glucose) OR (canagliflozin) OR (Ipragliflozin) OR (Sodium-Glucose Cotransporter) OR (Luseogliflozin) OR (SGLT2) OR (ertugliflozin) OR (tofogliflozin) OR (remogliflozin) OR (sodium glucose cotransporter 2)) – 08/10/2024, zero results

SGLT2 inhibitors in Fluoropyrimidine-induced cardiotoxicity

((5FU) OR (5-FU) OR (Fluoropyrimidine) OR (Capecitabine)) AND ((empagliflozin) OR (dapagliflozin) OR (SGLT2i) OR (Sodium-Glucose) OR (canagliflozin) OR (Ipragliflozin) OR (Sodium-Glucose Cotransporter) OR (Luseogliflozin) OR (SGLT2) OR (ertugliflozin) OR (tofogliflozin) OR (remogliflozin) OR (sodium glucose cotransporter 2)) – 08/10/2024, eight results, one relevant

SGLT2 inhibitors in VEGFi-induced cardiotoxicity

((vascular endothelial growth factor inhibitor) OR (VEGF) OR (VEGF-i) OR (VEGFi) OR (aflibercept) OR (bevacizumab) OR (ramucirumab) OR (axitinib) OR (cabozantinib) OR (lenvatinib) OR (pazopanib) OR (regorafenib) OR (sorafenib) OR (sunitinib) OR (vandetanib)) AND ((empagliflozin) OR (dapagliflozin) OR (SGLT2i) OR (Sodium-Glucose) OR (canagliflozin) OR (Ipragliflozin) OR (Sodium-Glucose Cotransporter) OR (Luseogliflozin) OR (SGLT2) OR (ertugliflozin) OR (tofogliflozin) OR (remogliflozin) OR (sodium glucose cotransporter 2)) – 08/10/2024, 78 results, two relevant, including one previously found relevant article

SGLT2 inhibitors in BTK-induced cardiotoxicity

((Ibrutinib) OR (BTK) OR (Bruton Tyrosine Kinase) OR (BTK-inhibitors) OR (BTK-i) OR (Bruton Tyrosine Kinase inhibitor)) AND ((empagliflozin) OR (dapagliflozin) OR (SGLT2i) OR (Sodium-Glucose) OR (canagliflozin) OR (Ipragliflozin) OR (Sodium-Glucose Cotransporter) OR (Luseogliflozin) OR (SGLT2) OR (ertugliflozin) OR (tofogliflozin) OR (remogliflozin) OR (sodium glucose cotransporter 2)) – 08/10/2024, eight results, zero relevant

SGLT2 inhibitors in CAR-T-induced cardiotoxicity

((CAR-T) OR (Chimeric antigen receptor T cell) OR (Chimeric antigen receptor)) AND ((empagliflozin) OR (dapagliflozin) OR (SGLT2i) OR (Sodium-Glucose) OR (canagliflozin) OR (Ipragliflozin) OR (Sodium-Glucose Cotransporter) OR (Luseogliflozin) OR (SGLT2) OR (ertugliflozin) OR (tofogliflozin) OR (remogliflozin) OR (sodium glucose cotransporter 2)) – 08/10/2024, one result, zero relevant

SGLT2 inhibitors in radiotherapy-induced cardiotoxicity

((Radiation) OR (radiotherapy)) AND ((empagliflozin) OR (dapagliflozin) OR (SGLT2i) OR (Sodium-Glucose) OR (canagliflozin) OR (Ipragliflozin) OR (Sodium-Glucose Cotransporter) OR (Luseogliflozin) OR (SGLT2) OR (ertugliflozin) OR (tofogliflozin) OR (remogliflozin) OR (sodium glucose cotransporter 2)) – 08/10/2024, 153 results, zero relevant after excluding duplicates

SGLT2 inhibitors in multiple myeloma therapy-induced cardiotoxicity

((Alkylating) OR (alkylating agents) OR (cyclophosphamide) OR (melphalan) OR (Lenalidomide) OR (Pomalidomide) OR (Thalidomide) OR (proteasome inhibitor) OR (Bortezomib) OR (carfilzomib) OR (daratumumab) OR (Elotuzumab) OR (Isatuximab)) AND ((empagliflozin) OR (dapagliflozin) OR (SGLT2i) OR (Sodium-Glucose) OR (canagliflozin) OR (Ipragliflozin) OR (Sodium-Glucose Cotransporter) OR (Luseogliflozin) OR (SGLT2) OR (ertugliflozin) OR (tofogliflozin) OR (remogliflozin) OR (sodium glucose cotransporter 2)) – 08/10/2024, 64 results, four relevant, three after excluding duplicates

SGLT2 inhibitors in rapidly accelerated fibrosarcoma and mitogen-activated extracellular signal-regulated kinase inhibitor treatment-induced cardiotoxicity

((RAF inhibitors) OR (vemurafenib) OR (dabrafenib) OR (encorafenib) OR (MEK inhibitors) OR (trametinib) OR (cobimetinib) OR (binimetinib) OR (selumetinib)) AND ((empagliflozin) OR (dapagliflozin) OR (SGLT2i) OR (Sodium-Glucose) OR (canagliflozin) OR (Ipragliflozin) OR (Sodium-Glucose Cotransporter) OR (Luseogliflozin) OR (SGLT2) OR (ertugliflozin) OR (tofogliflozin) OR (remogliflozin) OR (sodium glucose cotransporter 2)) – 08/10/2024, 17 results, zero relevant
